# Behavioral and Historical Processes Jointly Maintain Genetic Connectivity in Fragmented Chinese Water Deer Populations

**DOI:** 10.1002/ece3.73678

**Published:** 2026-06-16

**Authors:** Zongzhi Li, Peng Liu, Zhirong Zhang, Junda Chen, Zhensheng Liu, Liwei Teng

**Affiliations:** ^1^ College of Wildlife and Protected Areas Northeast Forestry University Harbin China; ^2^ China Conservation and Research Centre for the Giant Panda Chengdu China; ^3^ Sichuan Provincial Institute of Forestry and Grassland Inventory and Planning Chengdu China; ^4^ Key Laboratory of Conservation Biology, National Forestry and Grassland Administration Harbin China

**Keywords:** Chinese water deer, conservation genetics, female‐biased dispersal, genetic connectivity, habitat fragmentation

## Abstract

Habitat fragmentation poses a major threat to endangered species by isolating populations and disrupting gene flow. The vulnerable Chinese water deer ( *Hydropotes inermis* ) in northeastern China offers a critical case for understanding how fragmented landscapes influence genetic viability. Through noninvasive sampling of 286 individuals genotyped at 11 microsatellite loci and mitochondrial markers, we evaluated genetic diversity, structure, and dispersal dynamics. Although microsatellite diversity was moderate (mean *H*_e_ = 0.661), positive *F*_is_ values indicated inbreeding or nonrandom mating within local groups. Surprisingly, population genetic structure was extremely weak (overall *F*_st_ = 0.008), and contemporary gene flow remained high, revealing that connectivity persists despite extensive habitat fragmentation. Our analyses show that this connectivity is maintained primarily through female‐biased dispersal—a striking contrast to the male‐biased dispersal typical of most artiodactyls. Higher female assignment indices and a greater proportion of females identified as recent dispersers consistently supported this pattern. Mitochondrial data further suggested a historical population expansion, providing context for the current genetic composition. These findings indicate that female‐mediated movement functions as a natural genetic rescue mechanism, mitigating the genetic drift and inbreeding expected in small, isolated populations. From a conservation perspective, this behavioral adaptation is essential for sustaining population viability. Effective management should therefore prioritize the protection and restoration of habitat corridors that facilitate female movement, ensuring that natural dispersal processes continue to maintain genetic health and long‐term persistence of Chinese water deer in northeastern China.

## Graphical abstract

We challenge a major paradigm in mammalian ecology by demonstrating female‐biased dispersal in an artiodactyl, the endangered Chinese water deer. This key behavioral mechanism drives high gene flow across a fragmented landscape, effectively creating a genetic rescue effect that maintains population connectivity and counters inbreeding.

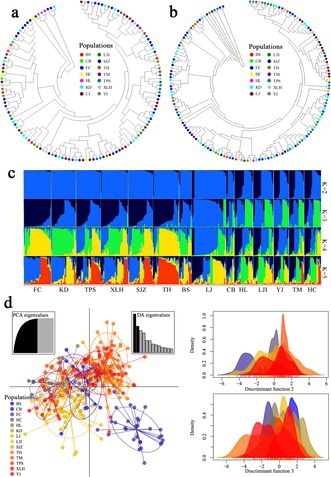

## 1 Introduction

The water deer ( *Hydropotes inermis* )—a vulnerable ungulate species recently rediscovered in northeastern China—faces ongoing pressures from human disturbance and habitat fragmentation (Li et al. 2023). Decades of poaching, habitat degradation, and land‐use change were thought to have extirpated the species from this region, making its reappearance a significant conservation opportunity (Z. Li 2024). To date, research on the water deer in northeast China has been limited to aspects such as distribution, diet, and habitat selection (Li et al. 2025). These studies confirm that the species persists in a human‐dominated landscape where remnant populations are likely isolated by roads, agriculture, and settlements. However, a critical knowledge gap remains: there is virtually no information on the population genetics, gene flow, or dispersal ecology of water deer in this fragmented setting (Li et al. 2025).

In broader conservation contexts, it is known that habitat fragmentation disrupts connectivity, impedes individual movement and gene flow, and can lead to genetic isolation, inbreeding, and erosion of genetic diversity (Wang et al. 2017; Connor et al. 2022; Fahrig 2003, 2019; Reed and Frankham 2003). Such genetic erosion diminishes evolutionary potential and population fitness, often triggering an “extinction vortex” (Frankham et al. 2017; Zhang et al. 2007). Conversely, maintaining or restoring functional connectivity has been shown to facilitate genetic exchange and can reverse genetic decline, as demonstrated in cases such as the Scandinavian wolf (Akesson et al. 2016; Clarke et al. 2024). For ungulates—a keystone group in forest ecosystems—population parameters such as density, genetic diversity, and dispersal patterns are essential for assessing ecosystem integrity, predator–prey dynamics, and vegetation renewal (Hu et al. 2024; Kerley et al. 2015; Chandru et al. 2020). Within the Northeast China Tiger and Leopard National Park, ungulates including red deer, roe deer, and wild boar form the primary prey base for the Amur tiger, with different species fulfilling distinct ecological niches that collectively support apex predator carrying capacity (Yang et al. 2018; Hojnowski et al. 2012). Yet, how these general principles apply to the recently rediscovered, presumably isolated water deer populations in northeastern China is entirely unexplored. Specifically, the role of water deer in local trophic networks and vegetation renewal cycles remains unquantified, and the genetic consequences of its prolonged isolation and recent population contraction are unknown (Lee and Lee 2020; Li et al. 2025).

The key information gap, therefore, lies in the absence of an integrated assessment of genetic diversity, population structure, and dispersal behavior in this threatened metapopulation. Without such data, it is impossible to evaluate whether current habitat fragments still support sufficient gene flow, or whether populations are already genetically depleted and structured by landscape barriers. Moreover, the dispersal tendencies of water deer—a life‐history trait critical for maintaining meta‐population connectivity—remain unstudied in this region, limiting evidence‐based corridor planning. Genetic diversity serves as a critical indicator of viability in endangered species, operating through dual mechanisms: high heterozygosity provides the foundation for adaptive evolution in response to environmental stressors (Robuchon et al. 2023), while allelic richness acts as a “genetic buffer” positively correlated with disease resistance and overall fitness (Margaryan et al. 2023). Population genetic structure accurately reflects the intensity of gene flow and historical divergence among local populations, offering a scientific basis for designing tailored conservation strategies—such as selecting individuals for genetic rescue and prioritizing corridor restoration (Opedal et al. 2017). Dispersal behavior further bridges genetic structure and ecological processes; as a key life‐history strategy, it facilitates movement across habitat patches, helps avoid inbreeding, expands mate choice, and allows access to new resources, thereby maintaining meta‐population level genetic diversity (Saastamoinen et al. 2018). An integrated research framework combining “genetic diversity, structure, and dispersal” is therefore urgently needed to address the conservation challenges faced by the water deer in northeastern China.

To address these gaps, this study aims to employ noninvasive genetic sampling to assess the current levels of genetic diversity, population genetic structure, and sex‐biased dispersal patterns of the water deer in northeastern China. Specifically, we hypothesize that: (1) habitat fragmentation and loss in northeastern China have resulted in limited gene flow among water deer populations, leading to inbreeding within populations; (2) due to small population sizes, inbreeding has reduced genetic diversity to relatively low levels; (3) physical barriers such as roads and geographical distance have contributed to genetic structuring among populations; and (4) the dispersal pattern of water deer is consistent with the male‐biased dispersal commonly observed in artiodactyls. By linking genetic metrics with landscape features, this work will provide a foundation for targeted conservation strategies—such as identifying genetic rescue candidates and prioritizing corridor restoration—to enhance the long‐term viability of this endangered ungulate in human‐modified landscapes.

## 2 Materials and Methods

### 2.1 Study Area

**FIGURE 1:**
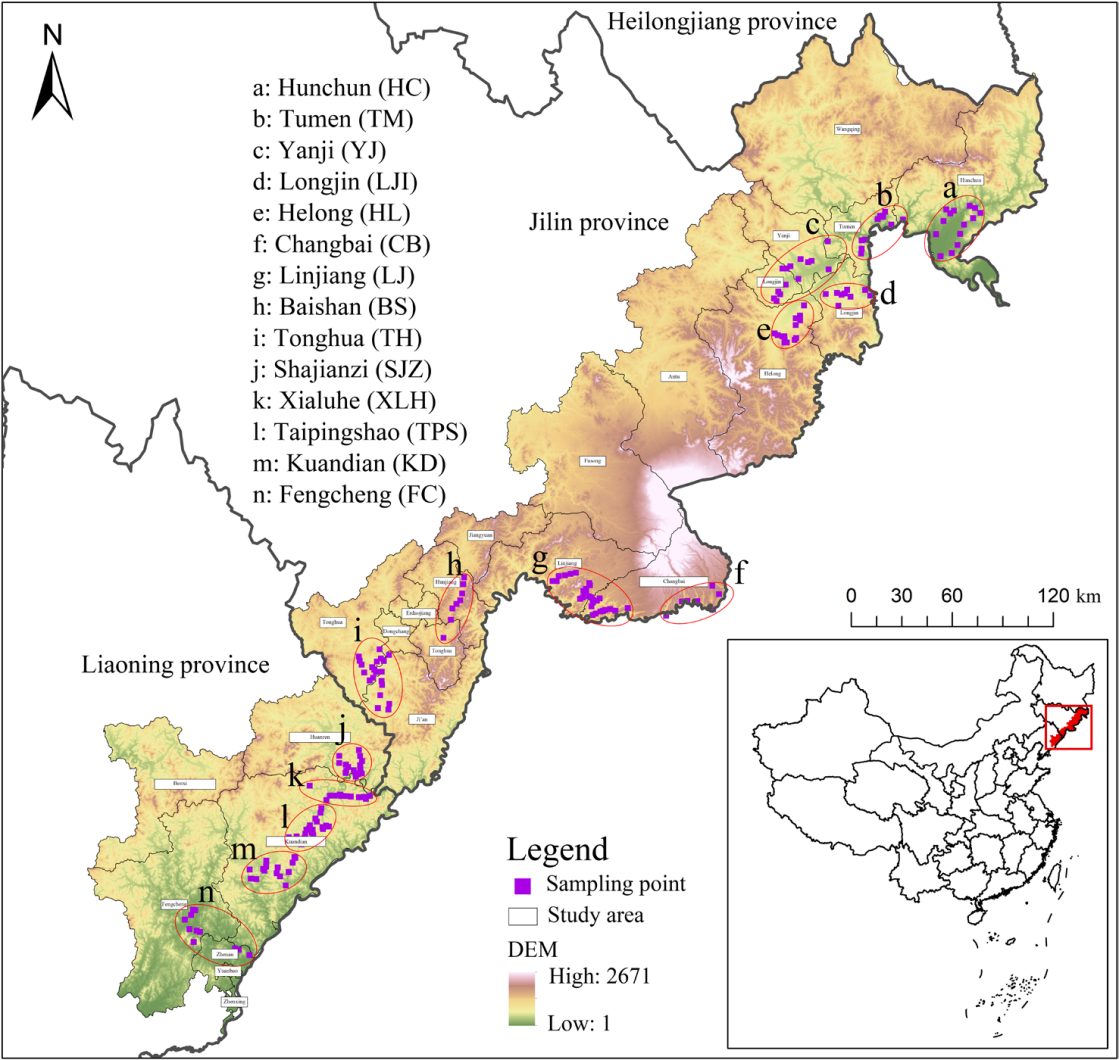
Study area map and geographic population division of samples. Red circles denote the 14 geographic populations of water deer labeled a‐n, which were delineated based on geographic distance and natural barriers.

This study was conducted in the Yalu River and Tumen River basins within the Changbai Mountain region of China, primarily covering parts of eastern Jilin Province and northeastern Liaoning Province (Figure 1). The study area extends from 123°33′ E to 131°19′ E and 40°2′ N to 44°3′ N, spanning approximately 440 km from north to south and 640 km from east to west. The central part of the region features the highest elevations, with the highest point being Baiyun Peak in the Changbai Mountain range at 2691 m (Z. Zhao 1999). From the central region toward the southwest, elevation gradually decreases, with Kuandian Manchu Autonomous County, Fengcheng City, and the urban area of Dandong being among the lowest‐lying zones. Toward the northeast, elevation also declines moderately but remains predominantly mountainous above 800 m, with only limited low‐elevation areas around Yanji, Helong, Longjing, and Hunchun cities. The region exhibits a typical temperate continental monsoon climate. Influenced by solar radiation, geographical location, and atmospheric circulation, it displays not only general mountainous climate characteristics but also distinct vertical climatic zonation, ranging from mid‐temperate and cold‐temperate to alpine sub‐frigid zones from lower to higher elevations (X. Li 2011).

### 2.2 Collection of Water Deer Feces Samples and DNA Extraction

From June 2021 to July 2023, fresh fecal samples of water deer were collected within suitable habitats (Yang et al. 2018) and potential distribution areas across the study region. The freshness of feces was determined based on gloss, color, and firmness, and only samples estimated to be within 3 days of deposition were collected. When fresh feces were identified, individual pellets were picked up using sterilized forceps, placed into 15 mL collection tubes, labeled, and immediately stored in dry ice‐cooled containers. Based on topographic conditions, landscape structure, and geographical distance among sampling locations, all fecal samples were assigned to 14 geographic populations: a: Hunchun (HC), b: Tumen (TM), c: Yanji (YJ), d: Longjing (LJI), e: Helong (HL), f: Changbai (CB), g: Linjiang (LJ), h: Baishan (BS), i: Tonghua (TH), j: Shajianzi (SJZ), k: Xialuhe (XLH), l: Taipingpao (TPS), m: Kuandian (KD), and n: Fengcheng (FC) (Figure 1).

DNA was extracted from fecal samples using the QIAamp Fast DNA Stool Mini Kit (50) (Qiagen, Germany). Primers for amplifying the mitochondrial Cyt *b* and D‐loop regions of the water deer were selected from Chen (2006), as listed in Table S1. Amplification was performed according to the PCR reaction system and conditions detailed in Tables S2 and S3, respectively. A negative control was included for each sample, and each reaction was repeated at least three times to ensure accuracy. The PCR products were sent to Sangon Biotech (Shanghai) for sequencing. Based on published microsatellite primers for water deer and related species (Yu et al. 2011; Lee et al. 2011; N. Zhao 2018), 21 primer pairs were initially selected for polymorphism screening. Preliminary screening was conducted using DNA extracted from muscle tissue, followed by secondary screening with DNA from fecal samples. Primers that consistently amplified fecal DNA and exhibited high polymorphism were chosen for subsequent experiments. Finally, 11 primer pairs were selected (Table S4). PCR amplification was carried out under the conditions specified in Tables S2 and S3, with a negative control for each sample and a minimum of three replicates per reaction to ensure reliability. The amplified products were sequenced by Sangon Biotech (Shanghai). For sex identification, primers targeting the SRY gene on the Y chromosome published by Han et al. (2009) were used, along with the BMC1009 primer targeting an autosome as an internal control in a multiplex PCR assay, performed according to Tables S2 and S3. To avoid interference between primers in multiplex PCR, additional singleplex amplifications were conducted using the SRY12 primer (Table S5).

### 2.3 Data Analysis

#### 2.3.1 Species and Sex Identification, and Individual Discrimination

Mitochondrial sequences were aligned via BLAST against the NCBI database. Sequences showing > 98% similarity with water deer were identified as originating from this species. For samples confirmed as water deer, sex was determined through three independent PCR amplifications. Samples showing SRY bands in at least two of the three replicates were identified as male; those showing no SRY or SRY12 bands but exhibiting BMC1009 bands in all three replicates were identified as female. Individual identification was performed using CERVUS 3.0 based on microsatellite genotyping results. Samples were considered to originate from the same individual if all genotyped microsatellite loci were identical, or if only a single allele at one locus differed (Gao 2020).

#### 2.3.2 Population Genetic Diversity

Mitochondrial raw sequences were manually proofread using Chromas 2.6.6 and EditSeq, then trimmed and assembled in SeqMan. Alignment of mitochondrial sequences was performed with Clustal X 2.1 (Larkin et al. 2007). The number of haplotypes (*H*), haplotype diversity (*H*_d_), nucleotide diversity (*P*_*i*_), number of variable polymorphic sites, and number of parsimony informative sites were calculated using DnaSP 6.12 (Rozas et al. 2017). Base composition and transition/transversion ratios were computed in MEGA 11 (Tamura et al. 2021).

Micro‐Checker 2.2.3 (Van Oosterhout et al. 2004) was used to test for the presence of null alleles or allelic dropout at microsatellite loci. Genepop 4.7 (Rousset 2017) was employed to assess deviations from Hardy–Weinberg equilibrium (HWE) and linkage disequilibrium (LD) across loci. Significance was tested using a Markov chain method, with *p*‐values corrected via the Bonferroni method. The number of alleles (*N*_a_), effective number of alleles (*N*_e_), observed heterozygosity (*H*_o_), expected heterozygosity (*H*_e_), and fixation index (*F*) were calculated using GenAIEx 6.51 (Smouse et al. 2017). Polymorphism information content (*PIC*) for microsatellite loci was estimated with CERVUS 3.0 (Tc 2007).

#### 2.3.3 Population Genetic Structure

Phylogenetic trees were constructed based on Cyt‐b and D‐loop sequences of the water deer using Bayesian Inference (BI) in MEGA X. Genetic differentiation indices (*F*_st_) among geographic populations were calculated using Arlequin 3.5, and gene flow (Number of Migrants per Generation, *N*_m_) was estimated via the formula *N*_m_ = (1 − *F*_st_)/(4*F*_st_). Analysis of Molecular Variance (AMOVA) was performed to assess the distribution of genetic variation within and among populations, with significance tested using 1000 permutations (Gao 2020). The best nucleotide substitution model was identified in MEGA 11 to compute genetic distances, and a maximum likelihood (ML) method was used to construct haplotype phylogenetic trees (Tian 2021).

For microsatellite data, overall and pairwise population genetic differentiation (*F*_st_) and gene flow (Nm) were computed using GenAIEx 6.51 (Smouse et al. 2017). AMOVA was applied to evaluate the partitioning of genetic variation within and among populations. The phylogenetic tree was constructed using the same methodology as applied to the mitochondrial data. Population genetic structure was inferred using a Bayesian clustering approach in STRUCTURE 2.3.4 (Pritchard et al. 2000). The number of population clusters (*K*) was set from 1 to 14, with a Markov chain Monte Carlo (MCMC) length of 100,000 iterations and a burn‐in of 10,000 iterations. Each *K* was run 10 times independently. The results were analyzed using Structure Harvester (Earl and VonHoldt 2012), and the optimal *K* was determined based on both *L*(*K*) and Δ*K*. The outcomes were averaged using CLUMPP 1.1.2 (Jakobsson and Rosenberg 2007). Additionally, discriminant analysis of principal components (DAPC), a nonparametric method, was employed to characterize the genetic structure and clustering patterns among geographic populations.

#### 2.3.4 Population Historical Dynamics

For mitochondrial DNA data, neutrality tests and mismatch distribution analysis were performed using DnaSP 6.12 (Rozas et al. 2017) to detect potential population expansion events. Neutrality tests, including Fu's Fs and Tajima's *D*, were applied to determine whether the population deviated significantly from neutral mutation. The values suggest historical population expansion if significantly negative, a bottleneck if positive (Liao et al. 2010). Mismatch distributions were evaluated based on Harpending's Raggedness Index (Hri) and the sum of squared deviations (SSD) to determine whether rapid expansion occurred. A unimodal mismatch distribution is indicative of population expansion, while a multimodal or ragged distribution suggests population stability (Rogers and Harpending 1992).

For microsatellite data, the software Bottleneck 1.2 (Piry et al. 1999) was used to test for recent bottleneck events under the stepwise mutation model (SMM), infinite allele model (IAM), and two‐phase model of mutation (TPM), along with a two‐tailed Wilcoxon signed‐rank test with 1000 iterations. A mode shift analysis was also conducted: an L‐shaped distribution of allele frequencies indicates mutation‐drift equilibrium, while a distorted L‐shape suggests a recent bottleneck event (Luikart and Cornuet 1998).

#### 2.3.5 Analysis of Dispersal

The *F*_st_ values between female and male water deer groups were compared based on microsatellite analysis. As microsatellites are biparentally inherited nuclear markers, alleles at all loci are transmitted to both sexes, diluting signals from dispersing individuals. Therefore, the sex with a bias toward dispersal is expected to show lower genetic differentiation among geographic populations than the more philopatric sex (Gao 2020).

Genetic distances between individuals within each geographic group were calculated separately for females and males based on microsatellite data using GenAIEx 6.51 (Smouse et al. 2017). An independent samples *t*‐test was applied to determine whether genetic distances differed significantly between sexes. A larger average genetic distance within the dispersing sex would support sex‐biased dispersal (Peakall and Beattie 1995).

Assignment tests were conducted using GenAIEx 6.51 (Smouse et al. 2017) to compute assignment indices (AIc) for each sex. A negative mean AIc value suggests a higher probability of dispersal for that sex, while a positive value indicates higher philopatry (Favre et al. 1997). In the distribution of assignment indices, a “long tail” toward negative values—where the number of individuals with strongly negative AIc significantly exceeds those with positive values—provides additional evidence of dispersal, with individuals in this tail considered potential dispersers (Mossman and Waser 1999).

Using GENECLASS 2.0 (Piry et al. 2004), a likelihood‐based assignment method was employed to identify recent dispersers based on microsatellite genotypes. The method of Rannala and Mountain, based on Bayesian criteria, was applied with a Monte Carlo resampling algorithm using 100,000 simulated individuals. A threshold of *p* = 0.01 was set; individuals with assignment probabilities below this value were classified as dispersers (Paetkau et al. 2004). For each disperser, the population with the highest assignment probability was designated as its putative origin, and the straight‐line distance from the sampling location to the geographic center of that population was calculated using ArcGIS 10.8.

## 3 Results

A total of 363 fresh fecal samples from water deer were collected in this study. Among these, Cyt‐b sequences were successfully amplified from 136 samples, and D‐loop sequences from 158 samples, yielding amplification success rates of 37.47% and 43.53%, respectively (Table 1). After sequencing and editing, the *Cyt b* sequences of the water deer were 1140 bp in length, with a transition/transversion bias (R) of 1.27 (accession number: PZ333625‐PZ333728). The D‐loop region was 801–863 bp long, with a transition/transversion bias (R) of 0.93. For microsatellite analysis, samples with successful amplification at more than 9 loci were retained, resulting in 336 samples with a success rate of 93.56%. Using CERVUS, 50 duplicate individuals were identified and removed, leaving 286 unique individuals—238 females and 48 males—corresponding to an overall female‐to‐male sex ratio of 4.958:1. Tests for Hardy–Weinberg equilibrium conducted in Genepop showed significant deviation from equilibrium at loci BM4107, Hi05, Hlat702, and Hlca1101 (Table S6). Hardy–Weinberg equilibrium (HWE) tests across all populations revealed that multiple loci in each population significantly deviated from HWE (Table S7).

**TABLE 1:** The feces sample collection and amplification information of water deer.

Population	Sample size	Number of successful amplifications			Amplification success rates			Number of individuals	Sex ratio
		Cyt *b*	D‐loop	Microsatellite	Cyt *b*	D‐loop	Microsatellite		
BS	15	9	3	14	60.00	20.00	93.33	12	10:2
CB	9	2	5	9	22.22	55.56	100.00	9	9:0
FC	31	23	29	31	74.19	93.55	100.00	23	14:9
HC	21	4	3	19	19.05	14.29	90.48	18	16:2
HL	22	3	3	21	13.64	13.64	95.45	21	18:3
KD	31	14	26	28	45.16	83.87	90.32	20	17:3
LJ	40	16	14	39	40.00	35.00	97.50	35	33:2
LJI	25	4	1	23	16.00	4.00	92.00	22	17:5
SJZ	32	16	16	28	50.00	50.00	87.50	20	17:3
TH	30	13	15	30	43.33	50.00	100.00	25	22:3
TM	17	3	3	17	17.65	17.65	100.00	15	13:2
TPS	37	13	24	28	35.14	64.86	75.68	23	17:6
XLH	32	14	15	31	43.75	46.88	96.88	27	21:6
YJ	21	2	1	18	9.52	4.76	85.71	16	14:2
Total	363	136	158	336	37.47	43.53	92.56	286	238:48

### 3.1 Genetic Signals of Historical Expansion

**FIGURE 2:**
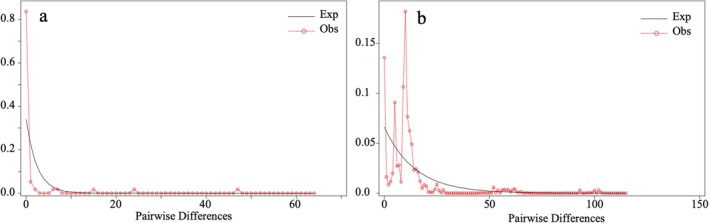
Mismatch distributions for water deer based mitochondrial data (Cyt *b*: a, D‐loop: b). The mismatch distribution of Cyt *b* exhibits a unimodal pattern, whereas that of D‐loop exhibits multimodal pattern.

The mismatch distribution based on Cyt *b* sequences displayed a unimodal pattern, suggesting that the water deer population in northeastern China may have undergone historical expansion. In contrast, the distribution derived from D‐loop sequences exhibited a multimodal curve, indicating a relatively stable demographic history (Figure 2). Neutrality tests including Tajima's *D* and Fu's Fs yielded the following values: for Cyt *b*, Tajima's *D* = −2.686 (*p* = 0.000), Fu's Fs = 0.492 (*p* = 0.643); for D‐loop, Tajima's *D* = −1.929 (*p* = 0.002), Fu's Fs = −2.563 (*p* = 0.312). The significantly negative Tajima's *D* values for both sequences provide evidence consistent with historical population expansion in the northeastern water deer population.

A total of eight haplotypes were identified from the Cyt *b* sequences, including 5 private haplotypes (62.50%). The number of haplotypes per population ranged from 1 to 4 (Table S8). There were 63 polymorphic sites, 50 parsimony informative sites. The haplotype diversity (*H*_d_) was 0.155 ± 0.042, and nucleotide diversity (*P*_*i*_) was 0.02554 ± 0.01060. For the D‐loop sequences, 42 haplotypes were defined, 26 of which were private (61.90%). The number of haplotypes per population ranged from 1 to 9 (Table S8). A total of 157 polymorphic sites and 82 parsimony informative sites were detected. The haplotype diversity (*H*_d_) was 0.864 ± 0.016, and nucleotide diversity (*P*_*i*_) was 0.02803 ± 0.00373. Results from the bottleneck analysis under three mutation models (IAM, TPM, SMM) indicated four instances of significant heterozygosity excess (*p* < 0.05) across geographic populations. These results suggest that the water deer populations in northeastern China likely have not experienced a recent decline (Table S9).

Genetic diversity analysis based on microsatellites revealed an average number of alleles (*N*_a_) of 6.617 and an average effective number of alleles (*N*_e_) of 4.036 across all geographic populations. Observed heterozygosity (*H*_o_) ranged from 0.449 (SJZ) to 0.678 (LJI), with an overall value of 0.584. Expected heterozygosity (*H*_e_) varied between 0.587 (KD) and 0.748 (YJ), with an overall value of 0.661. Analysis of inbreeding coefficients (*F*_is_) showed positive values in most populations, indicating the presence of nonrandom mating within these groups (Table 2).

**TABLE 2:** Genetic diversity of water deer based on microsatellites.

Poppulation	*N*_a_	*N*_e_	*H*_o_	*H*_e_	*F*_is_
BS	5.273	3.666	0.624	0.626	0.079
CB	5.273	3.984	0.559	0.628	0.091
FC	6.182	3.912	0.634	0.648	−0.006
HC	7.273	4.568	0.586	0.707	0.222
HL	7.727	4.672	0.638	0.747	0.150
KD	5.455	3.361	0.571	0.587	0.030
LJ	8.182	4.392	0.563	0.684	0.247
LJI	7.818	4.834	0.678	0.744	0.109
SJZ	5.636	3.299	0.449	0.602	0.226
TH	6.818	3.475	0.597	0.628	0.033
TM	5.909	3.584	0.575	0.651	0.140
TPS	6.091	3.677	0.538	0.628	0.114
XLH	6.636	3.932	0.544	0.630	0.156
YJ	8.364	5.146	0.624	0.748	0.183
Total	6.617	4.036	0.584	0.661	0.127

### 3.2 Population Genetic Structure

**FIGURE 3:**
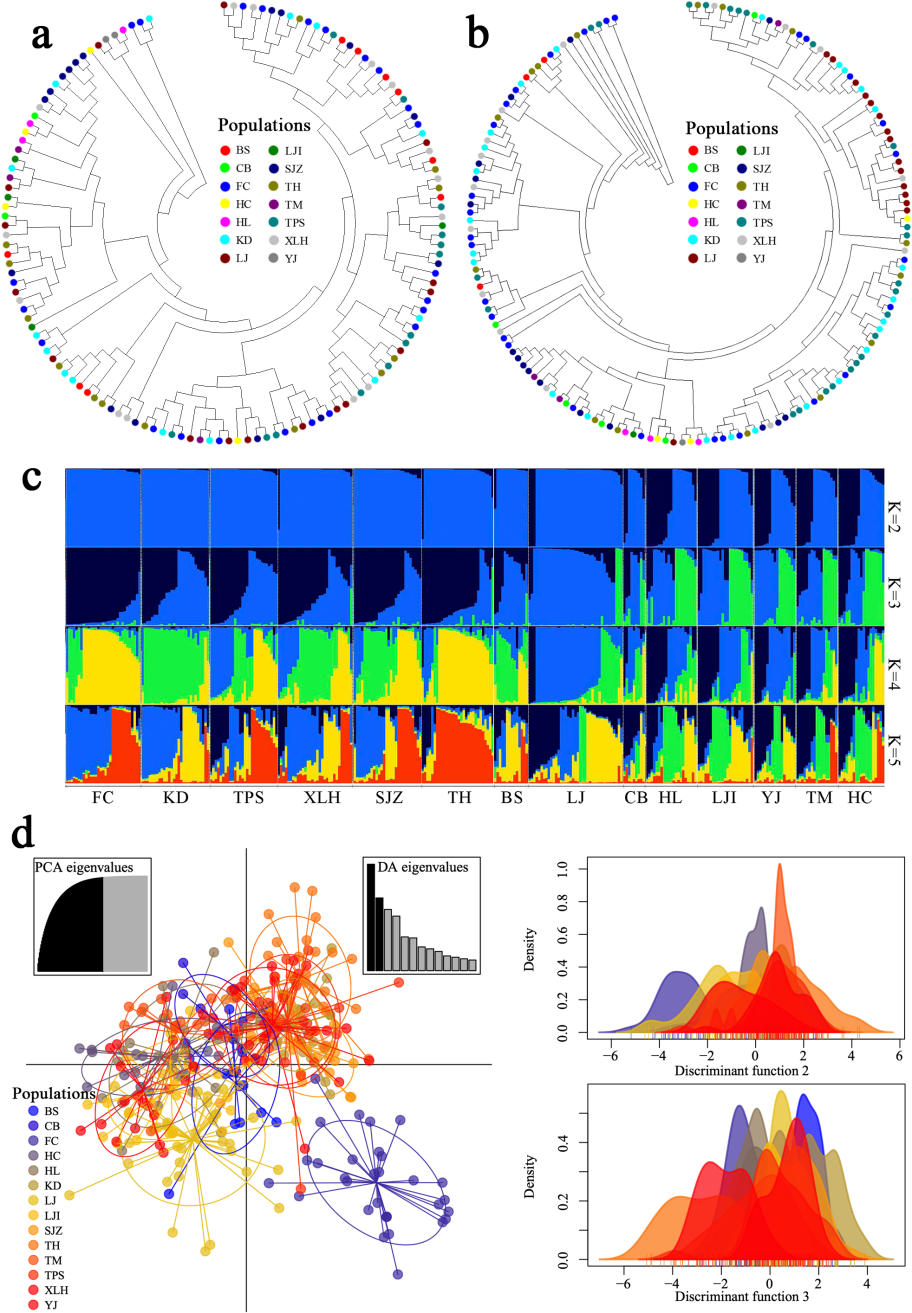
(a, b) The Neighbor‐joining (NJ) tree for water deer based on mitochondrial data (Cyt *b*: a, D‐loop: b). Dots of different colors represent samples from different water deer populations. Neither the Cyt *b* nor the D‐loop NJ tree shows clear population clustering; (c) STRUCTURE result of water deer based on microsatellite data. Each bar represents one individual. The different colors are the proportion of one individual assigned to a certain cluster; (d) Clustering of water deer based on principal component analysis and discriminant function. Geographic groups are indicated by ellipses, with dots representing individuals.

Phylogenetic trees constructed from mitochondrial *Cyt b* and D‐loop sequences using the neighbor‐joining method revealed no clear clustering pattern among geographic populations (Figure 3a,b). Individuals from various populations were intermingled throughout the tree, indicating a mixed clustering pattern and the absence of distinct population genetic structure. Haplotype networks based on *Cyt b* and D‐loop sequences showed no clear clustering or branching corresponding to populations (Figure S1).

Principal coordinate analysis based on microsatellite data at the individual level showed extensive intermixing among individuals from different geographic populations, with no obvious genetic structure (Figure S2). Similarly, a maximum likelihood phylogeny revealed no distinct clustering among geographic groups (Figure S3). STRUCTURE analysis indicated that the highest Δ*K* value occurred at *K* = 4 (Figure S4), dividing all individuals into four genetic clusters. Nearly all individuals were assigned to yellow, blue, green, and dark blue clusters (Figure 3c). Discriminant analysis of principal components (DAPC) showed that the centroids of most populations were closely grouped, with the exception of Fengcheng (FC), which was positioned farther from the center. Both discriminant functions supported this result, further confirming the lack of strong population genetic structure in the water deer (Figure 3d).

### 3.3 Molecular Evidence for Sex‐Biased Dispersal

**FIGURE 4:**
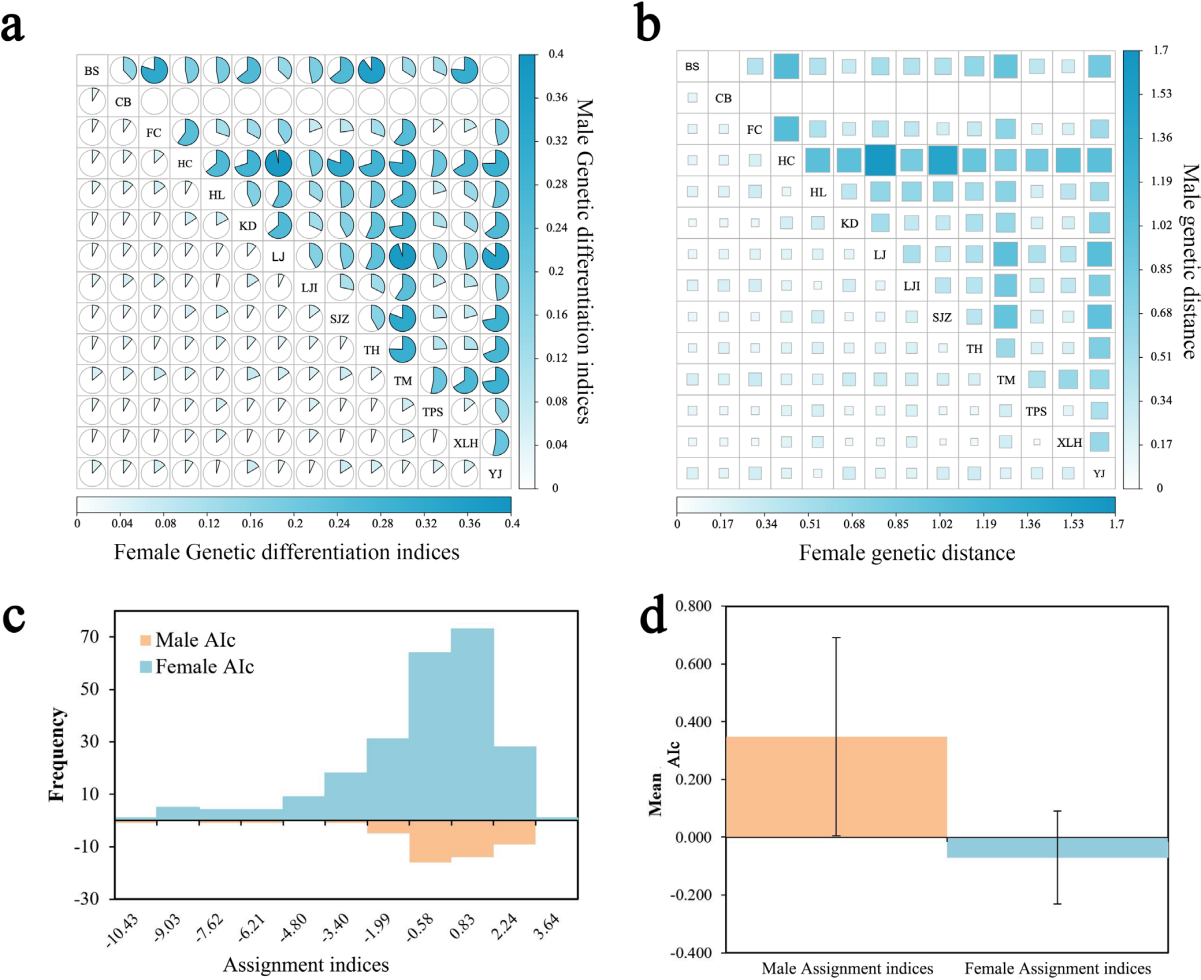
(a) Comparison of genetic differentiation indices of water deer between male and female. Values for males (upper‐right corner) are significantly larger than those for females (lower‐left corner); (b) Comparison of genetic distance of water deer between male and female. Values for males (upper‐right corner) are significantly larger than those for females (lower‐left corner); (c, d) AIc values of male and female water deer. AIc values are predominantly negative for both sexes, the mean AIc is negative for females but positive for males.

The average *F*_st_ value was significantly higher in males than in females (0.199 ± 0.009 vs. 0.043 ± 0.001; *t* = 17.743, *p* = 0.000), suggesting a female‐biased dispersal pattern (Figure 4a). In contrast, males had significantly higher genetic distances than females (0.581 ± 0.034 vs. 0.178 ± 0.006; *t* = 12.708, *p* = 0.000), which supports male‐biased dispersal (Figure 4b).

The majority of assignment indices (AIc) values were negative, indicating the occurrence of dispersal within the population. A long tail on the left side of the histogram—predominantly composed of females—suggests a higher incidence of dispersal among female individuals (Figure 4c). The mean AIc value was −0.070 for females and 0.348 for males; although the difference was not statistically significant (*Z* = 1.164, *p* = 0.244), this trend provides partial support for female‐biased dispersal.

Analysis using Geneclass detected 73 individuals (*p* < 0.01) that had undergone recent dispersal events (66 females, 27.73%; 7 males, 14.58%, Table S10). Dispersal distances ranged from 15.175 to 196.719 km (mean = 67.636 km) for females, and from 16.035 km to 104.015 km (mean = 51.136 km) for males. The difference in dispersal distance between sexes was not significant (*t* = 0.857, *p* = 0.394). These results indicate that both sexes disperse, with a slightly higher proportion of females engaging in dispersal behavior.

### 3.4 Coexistence Mechanism of High Gene Flow and Positive *F*_is_

AMOVA results from both mitochondrial (*Cyt b* and D‐loop) and microsatellite data showed that the vast majority of genetic variation (> 87%) resided within geographic populations, with only 4.65%–12.31% attributed to differences among populations (Tables S11 and S12).

**FIGURE 5:**
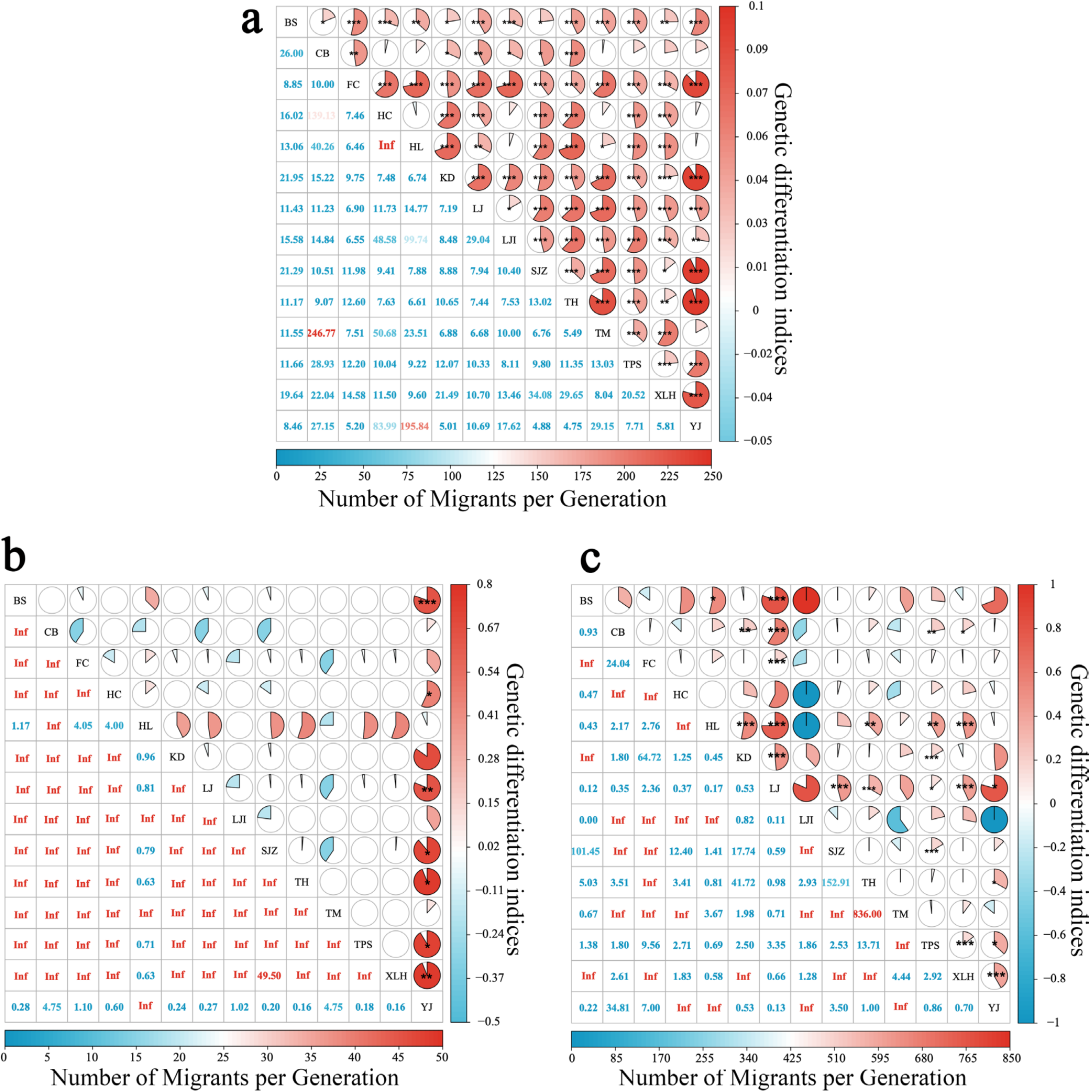
Genetic differentiation and gene flow between populations of water deer based on microsatellite and mitochondrial data. (a) Microsatellite data: genetic differentiation values range form −0.05 to 1, and gene flow values range from 0 to 250.(b) Cyt *b*: genetic differentiation values range from −0.5 to 0.8, and gene flow values range from 0 to infinity. (c) D‐loop: genetic differentiation values range from −1 to 1, and gene flow values range from 0 to infinity. Inf stands for infinity.

Fixation indices calculated from microsatellite data indicated low overall genetic differentiation (*F*_st_ = 0.008). The majority of loci showed positive values for both total inbreeding coefficient (*F*_it_) and within‐population inbreeding coefficient (*F*_is_), suggesting the presence of inbreeding or nonrandom mating within populations (Table 3). Pairwise *F*_st_ values between geographic populations showed that 78 pairs (85.71%) exhibited significant genetic differentiation (Figure 5). Among these, 33 population pairs (36.26%) showed moderate genetic differentiation (0.05 < *F*_st_ < 0.15), while the remaining pairs had *F*_st_ values below 0.05, indicating very low differentiation. Gene flow analysis confirmed ongoing genetic exchange among all geographic populations. The lowest level of gene flow (*N*_m_ = 4.75) was detected between TH and YJ, whereas an infinite *N*_m_ value between HL and HC indicated frequent genetic exchange between these two populations.

**TABLE 3:** *F*‐statistics and gene flows of different loci of water deer.

Locus	*F*_is_	*F*_it_	*F*_st_	*N*_m_
BM1706	−0.068	0.030	0.092	2.473
BM4107	0.496	0.524	0.055	4.328
Hi05	0.242	0.317	0.099	2.280
Hlat702	0.196	0.262	0.082	2.792
Hlca1101	0.206	0.250	0.055	4.261
Hlca1208	0.113	0.176	0.071	3.275
Hlca1501	0.018	0.060	0.043	5.564
Hlca1502	−0.016	0.083	0.098	2.303
Hlcal601	−0.017	0.046	0.062	3.783
Hlct710	−0.102	−0.059	0.039	6.128
T507	0.588	0.635	0.116	1.909
SE	0.068	0.065	0.008	0.421

Genetic differentiation analysis based on *Cyt b* sequences revealed infinite gene flow (*N*_m_) between 69 population pairs (75.82%), indicating frequent genetic exchange among these populations (Figure 5). Only seven population pairs (7.69%) showed significant genetic differentiation, with *F*_st_ values ranging from 0.455 (HC vs. YJ) to 0.752 (XLH vs. YJ), all exceeding 0.25 and thus classified as highly differentiated (Wright 1972). Analysis based on D‐loop sequences indicated infinite gene flow (*N*_m_) between 28 population pairs (30.77%), reflecting active genetic interchange among these groups (Figure 5). Significant genetic differentiation was detected in 24 population pairs (26.37%), with *F*_st_ values ranging from 0.129 (LJ vs. TPS) to 0.809 (BS vs. LJ). Among these, 2 pairs showed moderate genetic differentiation (0.05 < *F*_st_ < 0.15), while 22 pairs exhibited high differentiation (0.15 ≤ *F*_st_ ≤ 0.25) (Wright 1972).

## 4 Discussion

### 4.1 Historical and Contemporary Genetic Dynamics

Our mitochondrial and microsatellite analyses jointly reveal a complex demographic history for water deer in northeastern China. *Cyt b* mismatch distributions and significantly negative Tajima's *D* values indicate historical population expansion, whereas the multimodal mismatch pattern of the D‐loop suggests demographic stability in more recent periods. These contrasting signals likely reflect the different evolutionary timescales captured by the two markers, with *Cyt b* retaining deeper historical signatures and the rapidly evolving D‐loop reflecting more contemporary dynamics (Crochet and Desmarais 2000; Krojerová‐Prokešová et al. 2013). Such discrepancies are common in mammals due to differences in mutation rates and selective constraints across mitochondrial regions. Microsatellite‐based bottleneck tests revealed no evidence of recent population decline, and allele frequency distributions exhibited a typical L‐shaped pattern, suggesting mutation–drift equilibrium. These findings imply that water deer may have persisted continuously in northeastern China rather than experiencing local extirpation, despite decades of anthropogenic pressure. Field interviews further support this interpretation, with residents reporting frequent sightings over multiple decades. The species' high reproductive potential—early sexual maturity and large litter sizes—may have buffered populations against environmental fluctuations and human disturbance.

Genetic diversity indices reinforce this interpretation. Although haplotype diversity (*H*_d_) was lower than that of southern Chinese and reintroduced Shanghai populations (Table S13), nucleotide diversity (*P*_*i*_) was comparatively high, indicating substantial genetic variation within the northeastern population. This pattern mirrors observations in other ungulates, such as red deer and roe deer in Northeast China (Neigel and Avise 1993). Microsatellite results further support the presence of relatively high genetic diversity, with a mean PIC of 0.6827—higher than that reported for reintroduced populations (N. Zhao 2018). However, the lower effective number of alleles (*N*_e_) relative to the total number of alleles (*N*_a_) suggests that rare alleles may be at risk of loss, especially under continued habitat fragmentation (Tian 2008). Positive inbreeding coefficients (*F*_is_) across most populations indicate that nonrandom mating is occurring despite high overall gene flow.

### 4.2 Weak Genetic Structure and Drivers of High Connectivity

Across all analyses, water deer in northeastern China exhibited weak population genetic structure. Phylogenetic trees, haplotype networks, PCoA, and maximum‐likelihood clustering all showed extensive intermixing among populations, with no clear geographic clustering. STRUCTURE analysis suggested a weak division into two broad clusters corresponding to populations on either side of the Changbai Mountains, but this pattern was not strongly supported by other methods. DAPC results similarly showed tightly clustered population centroids, except for the Fengcheng population, which exhibited slight differentiation. These findings contradict our first and third hypotheses, which predicted limited gene flow and genetic structuring due to habitat fragmentation and physical barriers. Instead, gene flow appears extensive across the study area, with mitochondrial markers indicating infinite Nm values for many population pairs and microsatellites showing low overall *F*_st_ (0.008). AMOVA results further confirm that most genetic variation occurs within populations rather than among them.

The high connectivity observed is likely driven by several ecological and landscape factors. As noted in our results, “within the study area, with the exception of the high‐altitude Changbai Mountain region, there are no major mountain ranges or large ravines that could act as significant geographical barriers to gene flow… allowing free movement and preventing distinct clustering.” This landscape permeability facilitates dispersal and reduces the likelihood of genetic isolation. Additionally, Geneclass analysis detected recent dispersal events in 73 individuals (25.52%), with dispersal distances exceeding 100 km in some cases. Such long‐distance movements can homogenize genetic variation across populations. Differences in evolutionary rates between mitochondrial and microsatellite markers likely explain discrepancies in differentiation patterns. Microsatellites, with their high mutation rates, are more sensitive to recent demographic processes and can detect subtle differentiation even under high gene flow (Freeland 2020). In contrast, mitochondrial markers evolve more slowly and may underestimate recent divergence. Overall, the weak genetic structure observed suggests that landscape connectivity remains functionally intact despite human‐modified habitats.

### 4.3 Evolutionary Drivers and Interpretation of Sex‐Biased Dispersal

Our fourth hypothesis predicted male‐biased dispersal, consistent with patterns observed in many artiodactyls. However, the results were mixed and do not support a clear conclusion. *F*_st_ values were significantly higher in males than in females, suggesting lower male dispersal and thus female‐biased dispersal. Conversely, genetic distances were higher in males, which would support male‐biased dispersal. Assignment index distributions showed a long left tail dominated by females, indicating that females may disperse more frequently. Geneclass analysis detected a higher proportion of dispersing females (27.73%) than males (14.58%), although dispersal distances did not differ significantly between sexes. These conflicting results highlight the complexity of inferring sex‐biased dispersal from genetic data. In many ungulates, male‐biased dispersal is driven by competition for mates and territoriality, whereas females tend to be more philopatric. However, water deer exhibit unique behavioral traits—such as solitary habits and weak territoriality—that may reduce sex differences in dispersal. Additionally, environmental factors such as habitat fragmentation and resource distribution can influence dispersal strategies in both sexes.

A major confounding factor in our study is the highly skewed sex ratio (4.958 females per male). Such imbalance can bias dispersal metrics, particularly those based on assignment indices and *F*_st_ comparisons. For example, if males are underrepresented in the sample, estimates of male genetic structure may be inflated, leading to erroneous conclusions about dispersal patterns. Noninvasive sampling may also disproportionately capture females if their fecal deposition behavior differs from that of males. Given these limitations, we cannot conclusively determine whether dispersal is male‐ or female‐biased. Instead, our findings suggest that both sexes disperse, with females potentially playing a more prominent role in maintaining connectivity. This interpretation aligns with the high overall gene flow observed across the study area and the absence of strong genetic structure.

### 4.4 Reconciling High Gene Flow With Evidence of Nonrandom Mating

One of the most intriguing findings of this study is the coexistence of high gene flow and evidence of nonrandom mating within populations. Although gene flow estimates indicate frequent genetic exchange among populations, positive *F*_is_ values and deviations from Hardy–Weinberg equilibrium at several loci and in most populations suggest nonrandom mating within populations. This pattern may arise from demographic processes such as high local densities, limited mate choice within patches, or social structure. High gene flow and nonrandom mating can coexist when dispersal occurs primarily among a subset of individuals, while others remain philopatric. For example, if dispersal is driven by females while males remain in natal areas—or vice versa—family groups (parental nuclei) or nonrandom mating may persist despite regional connectivity. Additionally, gene flow estimates based on mitochondrial markers may reflect historical rather than contemporary processes, whereas microsatellites capture more recent demographic events.

Another explanation is that landscape connectivity may be sufficient to allow movement but insufficient to ensure effective gene flow. For instance, individuals may disperse across the landscape but fail to reproduce successfully in new populations due to behavioral or ecological barriers. Alternatively, high local densities may increase the likelihood of mating among relatives, even when dispersal occurs. The AMOVA results, showing that most genetic variation occurs within populations, support the interpretation that inbreeding is occurring locally despite regional connectivity. Microsatellites, with their rapid mutation rates, may detect recent inbreeding events that mitochondrial markers cannot. Similar patterns have been observed in other ungulates, where high mobility coexists with fine‐scale genetic structuring. These findings underscore the importance of maintaining fine‐scale habitat connectivity and monitoring demographic processes. Conservation strategies should aim to reduce local inbreeding risk while preserving regional gene flow.

### 4.5 Conservation Implications and Recommendations

Our findings have several implications for conservation. First, the relatively high genetic diversity and extensive gene flow provide a strong foundation for long‐term population viability. However, signs of nonrandom mating and allele loss indicate emerging risks that require proactive management to prevent future genetic erosion and potential inbreeding. Maintaining and enhancing habitat connectivity—particularly across the Changbai Mountain region—should be a priority. Second, the skewed sex ratio warrants further investigation, as it may influence dispersal dynamics and effective population size. Finally, continued genetic monitoring is essential. Integrating mitochondrial, microsatellite, and genomic data will provide a more comprehensive understanding of demographic trends and help guide adaptive management strategies. By linking genetic patterns with landscape features, this study provides a foundation for evidence‐based conservation planning aimed at ensuring the long‐term persistence of water deer in northeastern China.

## Author Contributions

**Zongzhi Li:** conceptualization (lead), data curation (lead), investigation (lead), methodology (lead), resources (equal), software (equal), visualization (equal), writing – original draft (lead). **Peng Liu:** conceptualization (equal), data curation (equal), formal analysis (equal), resources (supporting), software (supporting), validation (supporting). **Zhirong Zhang:** conceptualization (equal), data curation (equal), formal analysis (equal), investigation (supporting), methodology (supporting), project administration (supporting), supervision (supporting), writing – review and editing (supporting). **Junda Chen:** conceptualization (equal), data curation (equal), formal analysis (equal), investigation (supporting), validation (supporting), writing – review and editing (supporting). **Zhensheng Liu:** conceptualization (equal), data curation (equal), formal analysis (equal), funding acquisition (lead), investigation (supporting), supervision (supporting), validation (supporting), writing – original draft (supporting), writing – review and editing (supporting). **Liwei Teng:** conceptualization (equal), data curation (equal), formal analysis (equal), funding acquisition (lead), investigation (supporting), methodology (equal), software (supporting), validation (supporting), writing – original draft (supporting), writing – review and editing (lead).

## Funding

This research was funded by “National Natural Science Foundation of China” (grant nos. 32071649, 32070519).

## Conflicts of Interest

The authors declare no conflicts of interest.

## Supporting information


**Table S1:** The information of mitochondrial DNA primer.
**Table S2:** PCR amplification system of water deer for mtDNA, microsatellite, and sex identification.
**Table S3:** PCR amplification reaction conditions.
**Table S4:** The information of microsatellite primer.
**Table S5:** The information of sex identification primer.
**Table S6:** The amplification information of microsatellite primers.
**Table S7:** Hardy–Weinberg equilibrium (HWE) tests for each population (*** indicating significant deviations from HWE).
**Table S8:** The distribution of haplotypes in the Cyt b and D‐loop data of water deer.
**Table S9:** Results of bottleneck effect test of water deer based on microsatellite data.
**Table S10:** Migrant detection of water deer.
**Table S11:** AMOVA analysis based on Mitochondrial Cyt b and D‐loop.
**Table S12:** Analysis of molecular variance (AMOVA) of different water deer population base on microsatellite.
**Table S13:** Comparison of genetic diversity of water deer and closely related species.
**Figure S1:** The haplotype network of water deer based on mitochondrial data (*Cyt b*: a, D‐loop: b).
**Figure S2:** PCoA of water deer based on microsatellite data.
**Figure S3:** Maximum Likelihood (ML) tree for water deer based on microsatellite data.
**Figure S4:** The Δ*K* plot for STRUCTURE analysis.

## Data Availability

The cyt b sequences used in this study have been deposited in GenBank under accession numbers PZ333625–PZ333728. All other required data are available as Supporting Information.
