# Supplementary material for: Behavioral and Historical Processes Jointly Maintain Genetic Connectivity in Fragmented Chinese Water Deer Populations
Source: Ecol Evol. 2026 Jun 16;16(6):e73678. doi: 10.1002/ece3.73678 (PMC13270403; doi:10.1002/ece3.73678)
Supplement: Supplementary file 1 — Table S1: The information of mitochondrial DNA primer. Table S2: PCR amplification system of water deer for mtDNA, microsatellite, and sex identification. Table S3: PCR amplification reaction conditions. Table S4: The information of microsatellite primer. Table S5: The information of sex identification primer. Table S6: The amplification information of microsatellite primers. Table S7: Hardy–Weinberg equilibrium (HWE) tests for each population (*** indicating significant deviations from HWE). Table S8: The distribution of haplotypes in the Cyt b and D‐loop data of water deer. Table S9: Results of bottleneck effect test of water deer based on microsatellite data. Table S10: Migrant detection of water deer. Table S11: AMOVA analysis based on Mitochondrial Cyt b and D‐loop. Table S12: Analysis of molecular variance (AMOVA) of different water deer population base on microsatellite. Table S13: Comparison of genetic diversity of water deer and closely related species. Figure S1: The haplotype network of water deer based on mitochondrial data (Cyt b: a, D‐loop: b). Figure S2: PCoA of water deer based on microsatellite data. Figure S3: Maximum Likelihood (ML) tree for water deer based on microsatellite data. Figure S4: The ΔK plot for STRUCTURE analysis. [file ECE3-16-e73678-s001.docx]

Table S1 The information of mitochondrial DNA primer

| Primer name | Sequence information (5’to3’) | T_A_ (℃) |
| --- | --- | --- |
| Cytb | F: GATATGAAAAACCATCGTTG  R: CCTTTTCTGGTTTACAAGAC | 45.8 |
| D-loop | F: GCATTATTAATATAGCTCCAT  AAAAACCAAGAACCTTGTTAG  R: TATAGATTTGAGTACAAAAA  ATGGCGCTTAAATACATACCT | 57.6 |

Table S2 PCR amplification system of water deer for mtDNA, microsatellite, and sex identification

| Reagent | mtDNA and sex identification | | Microsatellite | |
| --- | --- | --- | --- | --- |
|  | Concentration | Volume (μL) | Concentration | Volume (μL) |
| Template DNA | 40 ng | 2 | 20-50 ng/μl | 1 |
| Primers F | 10 μM | 0.75 | 10 μM | 0.5 |
| Primers R | 10 μM | 0.75 | 10μM | 0.5 |
| dNTPs | 10 mM | 5 | 5 μM | 0.5 |
| 2XPCR buffer | 10X | 12.5 | 10 X | 2.5 |
| Enzyme | KOD FX Neo 5 U/μL | 1 | Taq 5 U/μL | 0.2 |
| Add ddH2O to |  | 25 |  | 25 |

Table S3 PCR amplification reaction conditions

| Number | Operation | Temperature | Time |
| --- | --- | --- | --- |
| 1 | Pre-transformation | 95°C | 5 min |
| 2 | Transformation | 94°C | 30 sec |
| 3 | Annealing | 60°C | 30 sec |
| 4 | Extension | 72°C | 30 sec |
| 5 | Cycle 2-4 | 10 cycles |  |
| 6 | Transformation | 94°C | 30 sec |
| 7 | Annealing | 55°C | 30 sec |
| 8 | Extension | 72°C | 30 sec |
| 9 | Cycle 6-8 | 30 cycles |  |
| 10 | Repair extension | 72°C | 10 min |

Table S4 The information of microsatellite primer

| Primer | Sequence information (5’to3’) | T_A_(℃) | Fluorescent dye | Species origin |
| --- | --- | --- | --- | --- |
| Hi05 | F:CCATGTTGGACCCAGGAGTA  R:CCATCTGCCCAGATTTTCAT | 56.7 | HEX | Water deer |
| HIct710 | F:TGTAAAACGACGGCCAGTTGAAGTGGCAAACTCTAAGTGA  R:CAGCAGAAACTACACACAACATT | 53.4 | HEX | Water deer |
| HIcal601 | F:TGTAAAACGACGGCCAGTAAGATTCAGACAGGAAATGAGC  R:ATCAGCACCAGAGTCTCATAGG | 52.2 | FAM | Water deer |
| HIca1502 | F:TGTAAAACGACGGCCAGTAGCATAAGGGGGAAAAAGTTAC  R:CGGACACAACTGAAGTAGACTG | 52.2 | HEX | Water deer |
| HIcal501 | F:TGTAAAACGACGGCCAGTATCC ATTCATCCCAGGTTATAG  R:CAGGTGGATCACACAGTAAAGA | 50.9 | HEX | Water deer |
| HIca1208 | F:TGTAAAACGACGGCCAGTGAGTGAGAAAAGCAAAGCAGTC  R:GTGTCCTGCTCAACTTCACTCT | 54.1 | HEX | Water deer |
| HIca1101 | F:TGTAAAACGACGGCCAGTTCTCAGAAACTGTCACCA  R:GCCATATATCCAAGACCATC | 49.6 | FAM | Water deer |
| HIat702 | F:TGTAAAACGACGGCCAGTTCTTCCCAGTTAGTCCACAAGT  R:CAGAGACAAACATTAGGTCTTGTAAA | 55.2 | FAM | Water deer |
| T507 | F:AGGCAGATGCTTCACCATC  R:TGTGGAGCACCTCACACAT | 55.2 | FAM | Red deer |
| BM4107 | F:ATAGGCTTTGCATTGTTCAGG  R:AGCCCCTGCTATTGTGTGAG | 53 | FAM | Cattle |
| BM1706 | F:ACAGGACGGTTTCTCCTTATG  R:CTTGCAGTTTCCCATACAAGG | 54.1 | HEX | Cattle |

Table S5 The information of sex identification primer

| Primer name | Sequence information (5’to3’） | T_A_(℃) |
| --- | --- | --- |
| SRY | F: GCTGGGGTATGAGTGGAAAA  R: GTTTTCCGACGAGGTCGATA | 54.8 |
| BMC1009 | F: GCACCAGCAGAGAGGACATT  R: ACCGGCTATTGTCCATCTTG | 61 |
| SRY12 | F: CTTCATTGTGTGGTCTCGTG  R: CGGGTATTTGTCTCGGTGTA | 61 |

Table S6 The amplification information of microsatellite primers

| Locus | Number of alleles | Simple size | *H_o_* | *H_e_* | PIC | HWE |
| --- | --- | --- | --- | --- | --- | --- |
| BM1706 | 19 | 185 | 0.838 | 0.877 | 0.862 | NS |
| BM4107 | 15 | 254 | 0.421 | 0.865 | 0.849 | *** |
| Hi05 | 18 | 336 | 0.205 | 0.288 | 0.277 | *** |
| Hlat702 | 16 | 313 | 0.498 | 0.69 | 0.643 | *** |
| Hlca1101 | 16 | 330 | 0.621 | 0.835 | 0.815 | *** |
| Hlca1208 | 16 | 335 | 0.701 | 0.836 | 0.817 | NS |
| Hlca1501 | 23 | 336 | 0.795 | 0.84 | 0.821 | NS |
| Hlca1502 | 13 | 330 | 0.603 | 0.673 | 0.62 | NS |
| Hlcal601 | 10 | 335 | 0.773 | 0.84 | 0.82 | NS |
| Hlct710 | 9 | 336 | 0.827 | 0.785 | 0.754 | NS |
| T507 | 8 | 232 | 0.095 | 0.243 | 0.232 | NS |

Table S7 Hardy–Weinberg equilibrium (HWE) tests for each population (*** indicating significant deviations from HWE)

| Population | BS | CB | FC | HC | HL | KD | LJ | LJI | SJZ | TH | TM | TPS | XLH | YJ |
| --- | --- | --- | --- | --- | --- | --- | --- | --- | --- | --- | --- | --- | --- | --- |
| BM1706 | NS | NS | *** | NS | NS | *** | *** | *** | NS | NS | NS | *** | NS | NS |
| BM4107 | *** | *** | *** | *** | *** | *** | *** | *** | *** | *** | *** | *** | *** | *** |
| Hi05 | NS | NS | NS | *** | *** | NS | *** | *** | NS | NS | *** | NS | NS | *** |
| Hlat702 | *** | *** | *** | NS | NS | NS | *** | *** | *** | NS | *** | *** | *** | NS |
| Hlca1101 | NS | NS | *** | *** | *** | *** | *** | NS | *** | *** | *** | *** | *** | *** |
| Hlca1208 | NS | NS | NS | *** | *** | *** | *** | *** | *** | *** | NS | NS | NS | *** |
| Hlca1501 | NS | NS | *** | NS | *** | NS | *** | NS | *** | NS | NS | *** | NS | *** |
| Hlca1502 | NS | NS | *** | NS | NS | *** | NS | NS | NS | NS | *** | NS | NS | NS |
| Hlcal601 | NS | NS | *** | NS | *** | NS | NS | NS | *** | *** | NS | *** | NS | NS |
| Hlct710 | NS | NS | *** | NS | NS | NS | *** | NS | NS | NS | NS | *** | NS | *** |
| T507 | *** | NS | NS | NS | NS | NS | *** | *** | NS | NS | *** | NS | *** | *** |

Table S8 The distribution of haplotypes in the Cyt b and D-loop data of water deer

| Genes | Population | Number of haplotypes | Haplotype frequency (%) | Haplotype (Bold indicates unique haplotype) |
| --- | --- | --- | --- | --- |
| Cyt b | BS | 1 | 6.72 | Hap_1 |
|  | CB | 1 | 1.49 | Hap_1 |
|  | FC | 4 | 16.42 | Hap_1，Hap_3，**Hap_4**，Hap_5 |
|  | HC | 1 | 2.99 | Hap_1 |
|  | HL | 2 | 2.24 | Hap_1，**Hap_8** |
|  | KD | 2 | 10.45 | Hap_1，Hap_3 |
|  | LJ | 2 | 11.94 | Hap_1，**Hap_2** |
|  | LJI | 1 | 2.99 | Hap_1 |
|  | SJZ | 2 | 11.19 | Hap_1，Hap_5 |
|  | TH | 1 | 9.70 | Hap_1 |
|  | TM | 1 | 2.24 | Hap_1 |
|  | TPS | 1 | 9.70 | Hap_1 |
|  | XLH | 1 | 10.45 | Hap_1 |
|  | YJ | 2 | 1.49 | **Hap_6**，**Hap_7** |
| D-loop | BS | 1 | 2.08 | Hap_1 |
|  | CB | 4 | 3.47 | Hap_1，Hap_4，**Hap_13**，Hap_14 |
|  | FC | 9 | 18.06 | Hap_1，Hap_2，Hap_3，Hap_14，**Hap_15**，**Hap_16**，**Hap_17**，**Hap_18**，**Hap_19** |
|  | HC | 2 | 2.08 | Hap_3，Hap_5 |
|  | HL | 3 | 2.08 | Hap_26，**Hap_27**，**Hap_28** |
|  | KD | 7 | 17.36 | Hap_1，Hap_2，Hap_3，**Hap_20**，**Hap_21**，Hap_22，**Hap_23** |
|  | LJ | 7 | 9.72 | Hap_3，Hap_5，**Hap_6**，**Hap_7**，**Hap_10**，**Hap_11**，**Hap_12** |
|  | SJZ | 7 | 11.11 | Hap_1，Hap_2，Hap_3，Hap_4，Hap_22，**Hap_37**，Hap_38 |
|  | TH | 9 | 9.03 | Hap_1，Hap_2，Hap_3，Hap_4，Hap_38，**Hap_39**，**Hap_40**，**Hap_41**，**Hap_42** |
|  | TM | 2 | 2.08 | Hap_3，**Hap_24** |
|  | TPS | 5 | 11.81 | Hap_1，Hap_2，Hap_3，**Hap_33**，**Hap_34** |
|  | XLH | 6 | 9.72 | Hap_1，Hap_2，Hap_3，Hap_22，**Hap_35**，**Hap_36** |
|  | YJ | 2 | 1.39 | **Hap_25**，Hap_26 |

Table S9 Results of bottleneck effect test of water deer based on microsatellite data

| Population | Heterozygosity excess (*P* value) | | | Mode-shift |
| --- | --- | --- | --- | --- |
|  | IAM | TPM | SMM |  |
| BS | 0.231683 | 0.237176 | 0.290098 | Normal L-shaped |
| CB | 0.539603 | 0.429302 | 0.458806 | Normal L-shaped |
| FC | **0.015797** | 0.084579 | 0.307947 | Normal L-shaped |
| HC | 0.469617 | 0.508094 | 0.102589 | Normal L-shaped |
| HL | 0.480082 | 0.473074 | 0.138226 | Normal L-shaped |
| KD | 0.150604 | 0.363134 | 0.366869 | Normal L-shaped |
| LJ | 0.496406 | 0.512875 | **0.041694** | Normal L-shaped |
| LJI | 0.297507 | 0.518476 | 0.106636 | Normal L-shaped |
| SJZ | 0.12087 | 0.586859 | 0.395169 | Normal L-shaped |
| TH | 0.218463 | **0.040192** | **0.000082** | Normal L-shaped |
| TM | 0.200448 | 0.433982 | 0.327979 | Normal L-shaped |
| TPS | 0.097366 | 0.493052 | 0.256763 | Normal L-shaped |
| XLH | 0.082584 | 0.46438 | 0.280574 | Normal L-shaped |
| YJ | 0.110552 | 0.503638 | 0.108259 | Normal L-shaped |

Note: Bold indicates that the population may have bottleneck effect.


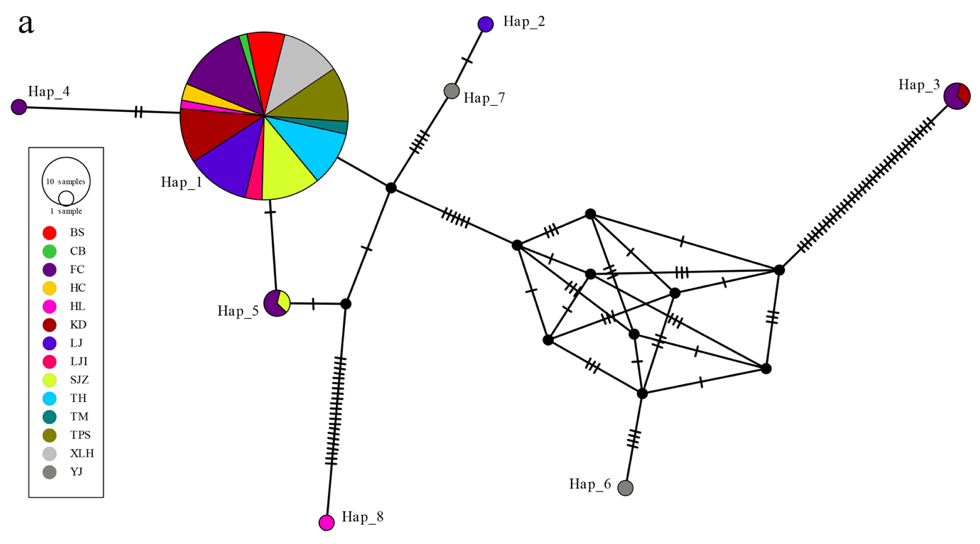

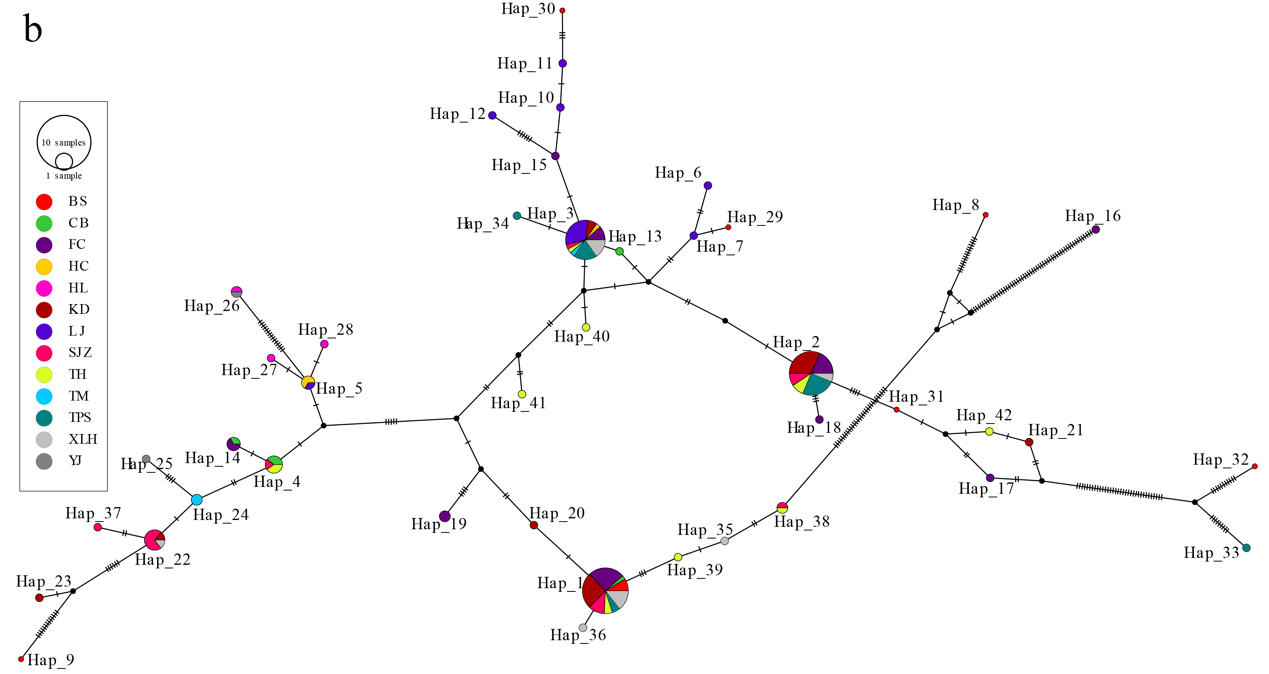


Figure S1 The haplotype network of water deer based on mitochondrial data (*Cyt* *b*: a, D-loop: b)


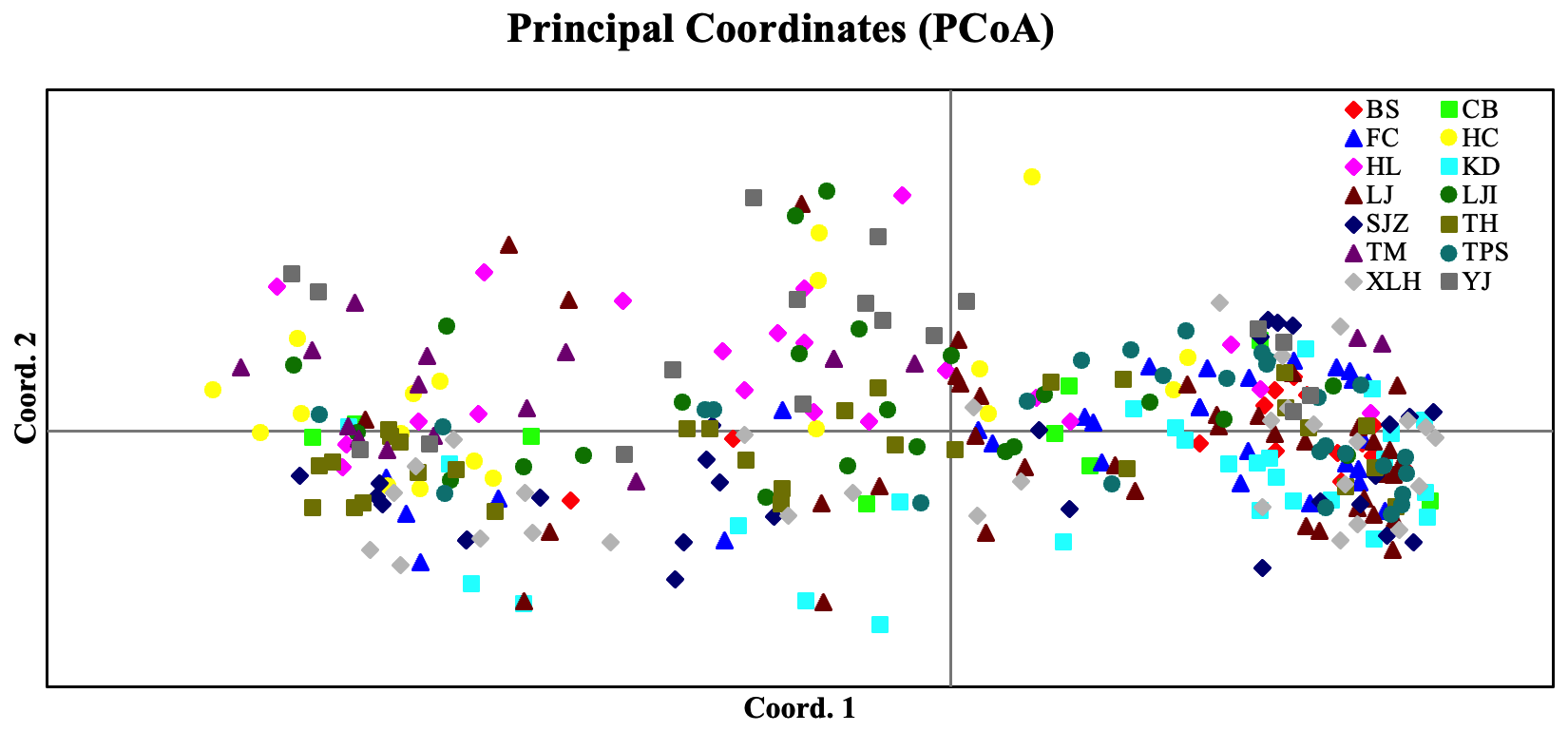


Figure S2 PCoA of water deer based on microsatellite data


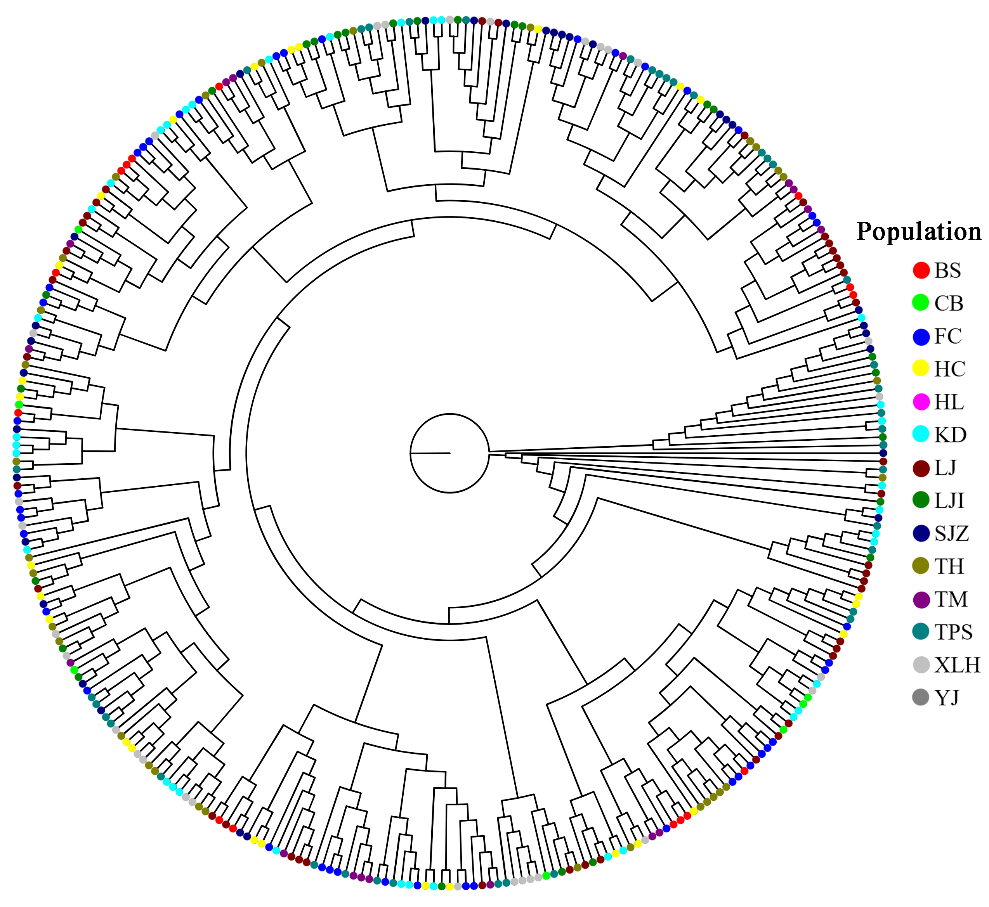


Figure S3 Maximum Likelihood (ML) tree for water deer based on microsatellite data


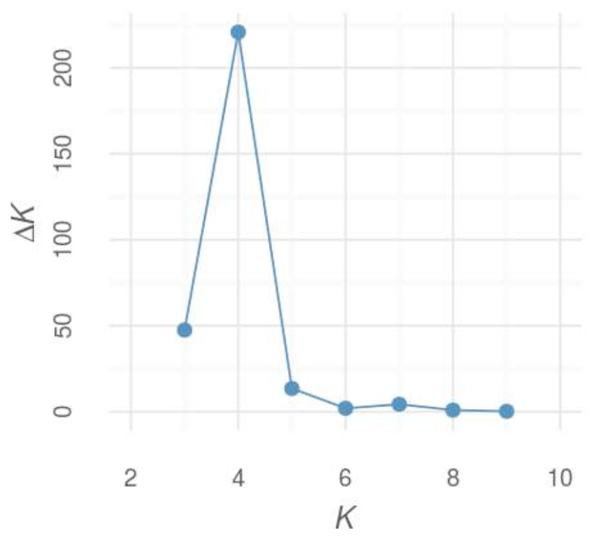


Figure S4 The ΔK plot for STRUCTURE analysis

Table S10 Migrant detection of water deer

| Assigned sample | Sex | Home | Probability | Population with highest probability | Dispersal distance (km) |
| --- | --- | --- | --- | --- | --- |
| Z0010 | Female | FC | 0.00003 | XLH | 113.434 |
| Z0023 | Female | FC | 0.00167 | TH | 175.522 |
| Z0038 | Female | FC | 0.00005 | TH | 177.743 |
| Z0045 | Female | KD | 0.00057 | TPS | 33.013 |
| Z0047 | Female | KD | 0.0002 | TPS | 37.447 |
| Z0072 | Female | KD | 0.00005 | TH | 115.963 |
| Z0080 | Female | TPS | 0.00438 | SJZ | 50.976 |
| Z0083 | Female | TPS | 0.00562 | XLH | 28.67 |
| Z0088 | Female | TPS | 0 | FC | 88.87 |
| Z0091 | Male | TPS | 0.00095 | XLH | 16.116 |
| Z0103 | Female | TPS | 0.00559 | SJZ | 38.52 |
| Z0106 | Female | TPS | 0 | XLH | 27.187 |
| Z0115 | Female | TPS | 0 | KD | 18.615 |
| Z0117 | Female | XLH | 0.00286 | TPS | 20.188 |
| Z0118 | Female | XLH | 0.00169 | SJZ | 22.578 |
| Z0119 | Female | XLH | 0.00007 | SJZ | 22.578 |
| Z0122 | Male | XLH | 0.00271 | KD | 53.115 |
| Z0125 | Female | XLH | 0.00001 | SJZ | 19.034 |
| Z0132 | Female | XLH | 0.00006 | TPS | 26.241 |
| Z0141 | Female | XLH | 0.00086 | TPS | 34.424 |
| Z0145 | Female | SJZ | 0.00183 | TH | 52.125 |
| Z0148 | Male | SJZ | 0.00137 | XLH | 17.016 |
| Z0149 | Female | SJZ | 0.00932 | XLH | 18.215 |
| Z0154 | Female | SJZ | 0.00465 | BS | 115.259 |
| Z0161 | Female | SJZ | 0.00297 | XLH | 15.175 |
| Z0162 | Female | SJZ | 0.00176 | LJ | 168.794 |
| Z0173 | Female | SJZ | 0.00001 | KD | 48.644 |
| Z0175 | Female | TH | 0.00015 | KD | 107.658 |
| Z0184 | Female | TH | 0.00132 | XLH | 54.257 |
| Z0189 | Female | TH | 0.00344 | FC | 190.371 |
| Z0190 | Female | TH | 0.00669 | TPS | 103.995 |
| Z0197 | Female | TH | 0.00802 | BS | 63.023 |
| Z0203 | Female | TH | 0.00699 | CB | 196.719 |
| Z0208 | Female | BS | 0.00139 | LJ | 75.353 |
| Z0218 | Female | BS | 0 | LJ | 83.778 |
| Z0219 | Female | BS | 0.00429 | XLH | 107.396 |
| Z0233 | Female | LJ | 0.00876 | BS | 73.655 |
| Z0234 | Female | LJ | 0.00247 | TH | 126.193 |
| Z0248 | Female | LJ | 0 | XLH | 176.054 |
| Z0255 | Female | LJ | 0.00196 | TH | 136.748 |
| Z0263 | Female | CB | 0 | LJ | 58.232 |
| Z0264 | Female | CB | 0 | BS | 147.091 |
| Z0273 | Female | HC | 0 | YJ | 101.075 |
| Z0276 | Female | HC | 0.0026 | YJ | 98.076 |
| Z0281 | Female | HC | 0.00398 | LJI | 63.686 |
| Z0289 | Female | HC | 0 | TM | 43.035 |
| Z0290 | Female | HC | 0.00998 | TM | 43.035 |
| Z0291 | Female | HC | 0.00006 | YJ | 85.672 |
| Z0292 | Male | HC | 0 | HL | 104.015 |
| Z0295 | Female | TM | 0.00084 | HL | 80.066 |
| Z0301 | Female | TM | 0.00001 | YJ | 51.75 |
| Z0308 | Female | TM | 0.00004 | YJ | 33.093 |
| Z0310 | Male | TM | 0 | HL | 56.401 |
| Z0311 | Female | YJ | 0.00177 | LJI | 19.682 |
| Z0314 | Female | LJI | 0.00492 | YJ | 25.57 |
| Z0319 | Female | LJI | 0.00032 | YJ | 32.574 |
| Z0320 | Female | LJI | 0.00589 | YJ | 32.574 |
| Z0324 | Female | LJI | 0.00003 | LJ | 21.694 |
| Z0340 | Female | YJ | 0.00993 | TM | 41.33 |
| Z0342 | Female | YJ | 0.00231 | TM | 43.428 |
| Z0345 | Female | YJ | 0.00001 | HC | 96.879 |
| Z0351 | Female | YJ | 0 | HL | 20.388 |
| Z0356 | Male | YJ | 0.0078 | HL | 16.035 |
| Z0358 | Female | YJ | 0 | HL | 26.393 |
| Z0364 | Female | HL | 0.00046 | YJ | 30.663 |
| Z0366 | Female | HL | 0.00409 | LJI | 37.188 |
| Z0374 | Female | HL | 0.00234 | TM | 83.045 |
| Z0377 | Female | HL | 0.00004 | YJ | 39.056 |
| Z0378 | Female | HL | 0.00431 | LJ | 43.046 |
| Z0380 | Female | HL | 0.00346 | YJ | 39.459 |
| Z0502 | Female | SJZ | 0.00419 | TPS | 42.661 |
| Z0716 | Male | TPS | 0.00251 | TH | 95.252 |
| Z0728 | Female | XLH | 0 | SJZ | 19.137 |

Table S11 AMOVA analysis based on Mitochondrial Cyt b and D-loop

| Genes | Source of variation | d.f. | Sum of squares | Variance components | Percentage of variation (%) |
| --- | --- | --- | --- | --- | --- |
| Cyt b | Among populations | 13 | 21.635 | 0.10609 Va | 10.4 |
|  | Within populations | 88 | 80.424 | 0.91391 Vb | 89.6 |
|  | Total | 101 | 102.059 | 1.02 | 100 |
|  | Fixation Index | *F_st_*: 0.10401 | | | |
| D-loop | Among populations | 13 | 146.433 | 0.75034 Va | 12.31 |
|  | Within populations | 102 | 544.98 | 5.34295 Vb | 87.69 |
|  | Total | 115 | 691.414 | 6.09329 | 100 |
|  | Fixation Index | *F_st_*: 0.12314 | | | |

Table S12 Analysis of molecular variance (AMOVA) of different water deer population base on microsatellite

| Source of variation | *d.f.* | Sum of squares | Variance components | Percentage of variation (%) |
| --- | --- | --- | --- | --- |
| Among populations | 13 | 103.98 | 0.1174 Va | 4.65 |
| Within populations | 658 | 1585.253 | 2.4092 Vb | 95.35 |
| Total | 671 | 1689.234 | 2.5266 | 100 |
| Fixation Index | *F_st_*: 0.04646 | | | |

Table S13 Comparison of genetic diversity of water deer and closely related species

| Species | Study area | Cyt b | | D-loop | | Microsatellite | |
| --- | --- | --- | --- | --- | --- | --- | --- |
|  |  | *H_d_* | *P_i_* | *H_d_* | *P_i_* | *H_o_* | *H_e_* |
| Water deer | Northeast of China | 0.155 ± 0.042 | 0.02554 ± 0.0106 | 0.864 ± 0.016 | 0.02803 ± 0.00373 | 0.584  (0.449-0.678) | 0.661  (0.587-0.748) |
| Water deer | South of China, South Korea | 0.959 ± 0.018 | 0.01339 ± 0.00292 | 0.983 ± 0.014 | 0.0174 ± 0.0023 | — | — |
| Water deer | Shanghai | 0.4673 | 0.0091 | 0.9794 | 0.00468 | 0.627  (0.167-0.957) | 0.626  (0.323-0.854) |
| Water deer | Zhoushan Archipelago | — | — | — | — | 0.912 | 0.826 |
| Water deer | South of China |  |  | 0.923 ± 0.025 | 0.01318 ± 0.00146 | 0.531  (0.429-0.651) | 0.662  (0.465-0.744) |
| Water deer | South Korea | — | — | — | — | 0.533 | 0.622 |
| Water deer | South Korea | — | — | — | — | 0.000-1.000 | 0.050-0.880 |
| Red deer | Northeast of China | 0.849 | 0.00678 | 0.877 | 0.02126 | 0.687  (0.644-0.725) | 0.619  (0.564-0.689) |
| Roe deer | Northeast of China | 0.840 ± 0.0003 | 0.00149 ± 0.00025 | — | — | 0.979 ± 0.008 | 0.776 ± 0.021 |
